# High-flux ptychographic imaging using the new 55 µm-pixel detector ‘Lambda’ based on the Medipix3 readout chip

**DOI:** 10.1107/S2053273314014545

**Published:** 2014-09-12

**Authors:** R. N. Wilke, J. Wallentin, M. Osterhoff, D. Pennicard, A. Zozulya, M. Sprung, T. Salditt

**Affiliations:** a University of Göttingen, Institute for X-ray Physics, Friedrich-Hund-Platz 1, 37077 Göttingen, Germany; b Deutsches Elektronen-Synchrotron DESY, Notkestrasse 85, 22607 Hamburg, Germany

**Keywords:** phase retrieval, ptychography, coherent diffractive imaging, semi-transparent central stop, semi-transparent beam stop, Lambda detector, Medipix3 chip, solar-cell nanowires

## Abstract

Suitable detection systems that are capable of recording high photon count rates with single-photon detection are instrumental for coherent X-ray imaging. The new single-photon-counting pixel detector ‘Lambda’ has been tested in a ptychographic imaging experiment on solar-cell nanowires using Kirkpatrick–Baez-focused 13.8 keV X-rays. Taking advantage of the high count rate of the Lambda and dynamic range expansion by the semi-transparent central stop, a high-dynamic-range diffraction signal covering more than seven orders of magnitude has been recorded, which corresponds to a photon flux density of about 10^5^ photons nm^−2^ s^−1^ or a flux of ∼10^10^ photons s^−1^ on the sample. By comparison with data taken without the semi-transparent central stop, an increase in resolution by a factor of 3–4 is determined: from about 125 nm to about 38 nm for the nanowire and from about 83 nm to about 21 nm for the illuminating wavefield.

## Abstract 2

The Large Area Medipix-Based Detector Array (Lambda) has been used in a ptychographic imaging experiment on solar-cell nanowires. By using a semi-transparent central stop, the high flux density provided by nano-focusing Kirkpatrick–Baez mirrors can be fully exploited for high-resolution phase reconstructions.

## 1. Introduction   

Coherent diffractive imaging (Miao *et al.*, 1999 ▶; Chapman & Nugent, 2010 ▶; Thibault & Elser, 2010 ▶) and, in particular, its variant ptychography (Faulkner & Rodenburg, 2004 ▶; Thibault *et al.*, 2008 ▶) needs to deal with diffraction patterns spanning orders of magnitude which can be problematic for current photon-counting detectors. In the case of ptychography, this high-dynamic-range problem is more accentuated because both the strong signal of the primary beam and the weak diffracted signal of the probed object need to be recorded. Intended as high-resolution X-ray imaging techniques, the potential resolution is considered to be limited by the invested dose (Howells *et al.*, 2009 ▶). Consequently, a high primary-beam intensity is needed to achieve a high resolution. Numerical phasing of the diffraction data thus crucially depends on modern detectors which provide a high dynamic range. An alleviation of the high-dynamic-range problem can be achieved by giving up strict photon counting. For instance, the MM-PAD (Vernon *et al.*, 2007 ▶; Tate *et al.*, 2013 ▶) achieved a count rate of 10^8^ photons pixel^−1^ s^−1^ at 8 keV X-rays in a recent ptychography study (Giewekemeyer *et al.*, 2014 ▶). Another important development in this direction is the Adaptive Gain Integrating Pixel Detector (Potdevin *et al.*, 2009 ▶). In addition to the high dynamic range, fast readout, small pixels and low noise are also important. Single-photon-counting pixel detectors such as Pilatus (Kraft *et al.*, 2009 ▶) and Maxipix (Llopart *et al.*, 2002 ▶) have the advantage of effectively zero readout noise and can reach maximum count rates in the range of 10^5^–10^7^ photons pixel^−1^ s^−1^ (Toyokawa *et al.*, 2010 ▶; Trueb *et al.*, 2012 ▶). The new Large Area Medipix-Based Detector Array (Lambda) is being developed (Pennicard & Graafsma, 2011 ▶; Pennicard *et al.*, 2011 ▶, 2012 ▶) in order to better meet experimental demands. Based on the Medipix3 chip (Ballabriga, Campbell *et al.*, 2011 ▶), the Lambda is constructed with two counters for deadtime-free readout. In the standard mode it can operate at 2000 frames s^−1^. In combination with its small pixel size and the increased maximum photon count rate of about 3 × 10^5^ photons s^−1^ pixel^−1^ (Ballabriga, Blaj *et al.*, 2011 ▶), in comparison to its predecessor with the Medipix2 chip, the Lambda thus becomes a valuable device in synchrotron applications such as ptychography. Another important detector development, albeit with a larger pixel area (factor 1.86), for single photon counting while framing at kHz rates is the 75 µm-pixel detector ‘Eiger’ (Dinapoli *et al.*, 2011 ▶; Johnson *et al.*, 2012 ▶) which, very recently, was successfully operated in a ptychographic imaging experiment (Guizar-Sicairos *et al.*, 2014 ▶).

In terms of robust and efficient coherent X-ray diffractive imaging (CDI) algorithms (Gerchberg & Saxton, 1972 ▶; Fienup, 1978 ▶, 1982 ▶; Sayre, 1980 ▶; Bauschke *et al.*, 2002 ▶; Elser, 2003 ▶; Giewekemeyer *et al.*, 2011 ▶; Rodriguez *et al.*, 2013 ▶) that iteratively invert oversampled diffraction patterns of non-crystalline specimens (Sayre, 1952 ▶; Miao *et al.*, 1998 ▶), the ptychographic method originating from the field of electron microscopy (Hoppe, 1969*a* ▶,*b* ▶; Hoppe & Strube, 1969 ▶; Nellist *et al.*, 1995 ▶; Nellist & Rodenburg, 1998 ▶) has stirred significant interest in the past (Faulkner & Rodenburg, 2004 ▶, 2005 ▶; Rodenburg *et al.*, 2007 ▶; Rodenburg, 2008 ▶; Thibault *et al.*, 2008 ▶). Ptychography uses the so-called overlap constraint: the object is successively illuminated in such a way that neighbouring illuminated spots share common regions of the object (Bunk *et al.*, 2008 ▶). Importantly, this overlap constraint is powerful enough to estimate not only the object but also the illumination function (probe) even in non-ideal experimental conditions, *e.g.* partial coherence, broad bandpass illumination or a thick object (Maiden & Rodenburg, 2009 ▶; Thibault *et al.*, 2009 ▶; Thibault & Menzel, 2013 ▶; Claus *et al.*, 2013 ▶; Edo *et al.*, 2013 ▶; Suzuki *et al.*, 2014 ▶; Batey *et al.*, 2014 ▶; Clark *et al.*, 2014 ▶; Enders *et al.*, 2014 ▶). Hence, ptychography is also an ideal tool for analysing X-ray wavefields (Schropp *et al.*, 2010 ▶; Kewish, Guizar-Sicairos *et al.*, 2010 ▶; Kewish, Thibault *et al.*, 2010 ▶; Hönig *et al.*, 2011 ▶; Vila-Comamala *et al.*, 2011 ▶; Wilke *et al.*, 2012 ▶; Schropp *et al.*, 2013 ▶; Huang *et al.*, 2013 ▶). However, achieving optimum resolution in ptychographic imaging remains a challenge. On the algorithmic side, the results can be improved by *e.g.* correcting for positional errors (Guizar-Sicairos & Fienup, 2008 ▶; Maiden *et al.*, 2012 ▶; Beckers *et al.*, 2013 ▶; Tripathi *et al.*, 2014 ▶) but on the experimental side this problem is currently mainly approached by increasing the fluence (*e.g.* Takahashi *et al.*, 2011 ▶, 2013 ▶; Guizar-Sicairos *et al.*, 2012 ▶; Holler *et al.*, 2012 ▶; Schropp *et al.*, 2012 ▶; Wilke *et al.*, 2013 ▶).

In this work, we investigate the performance of the Lambda detector in a ptychographic imaging experiment on solar-cell nanowires using 13.8 keV photons at the high-brilliance synchrotron radiation source PETRAIII. In order to make use of the full, highly coherent flux of the Kirkpatrick–Baez (KB) focus we test the Lambda in combination with the semi-transparent central stop (Wilke *et al.*, 2012 ▶). The ptychographic probe reconstruction enables us to monitor the systematic variation of the beam size due to a change of the numerical aperture in front of the Kirkpatrick–Baez (KB) mirrors. In addition, the fluence on the sample can be accurately determined which, in turn, is used to achieve a fluence-optimized setting. The paper closes with a short discussion of the results in view of the Lambda detector.

## 2. Materials and methods   

### 2.1. Experimental setup   

**Figure 1:**
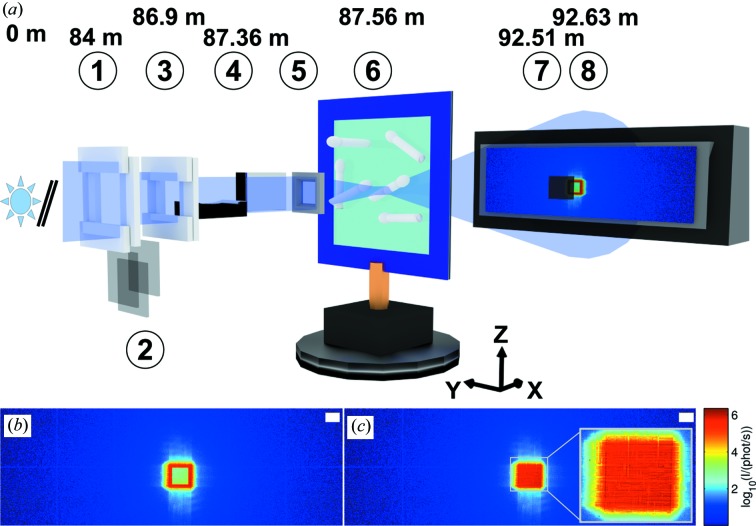
(*a*) Schematic of the GINIX setup: downstream of the undulator source and the monochromator (not shown) (1) slits S1, (2) attenuation foils made of Mo, (3) slits S2, (4) KB mirror system, (5) soft-edge aperture (∼12 mm in front of the focus), (6) nanowire sample in the focal plane, (7) STCS (Ge) and (8) Lambda detector. The flight tube between sample and detection device is not shown. (*b*) shows a diffraction pattern as recorded with the Lambda and the STCS (7). (*c*) Same diffraction pattern as in (*b*) but rescaled according to measured STCS-attenuation distribution. The colour bar is the same in (*b*) and (*c*). Scale bars in (*b*) and (*c*) denote 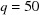
 µm^−1^.

The experiments with the Lambda detector were performed at the P10 coherence beamline of the PETRAIII synchrotron located at DESY, Hamburg in Germany. A monochromatic X-ray beam with an energy of 13.8 keV was selected by using a channel-cut monochromator. After passing a set of beam-defining slits, the beam was focused by a pair of X-ray mirrors in the KB geometry (*cf.* Fig. 1 ▶ *a*). The elliptically shaped mirrors (Pd coating) were used at 4.05 mrad incident angle (centre). The focal distances are 302 mm and 200 mm of the vertical mirror (WinlightX) and horizontal mirror (JTEC), respectively (for details see Kalbfleisch *et al.*, 2011 ▶; Kalbfleisch, 2012 ▶). A soft-edge, rectangular aperture was placed 12 mm upstream of the nominal focal plane to suppress side lobes of the X-ray beam (Takahashi *et al.*, 2013 ▶). The scanning of the sample through the X-ray beam was carried out by a high-precision piezo-electric stage (PI, Germany). Both the KB X-ray mirror system and the sample-positioning stages used are part of the Göttingen Instrument for Nano-Imaging with X-rays (GINIX). An evacuated flight tube of 5 m length was installed behind the sample stage to minimize scattering effects of X-rays in air. A positionable semi-transparent central stop (STCS) was installed in the flight tube. The STCS is a piece of germanium with lateral dimensions of about 4 × 4 mm, which was cut from a wafer using a dicing saw Disco dad 321 (Disco, Japan). Additionally, the Ge STCS was ground down to an average nominal thickness of 100 µm using SiC grinding paper with successive grain sizes 2000 and 4000 on a grinding machine LaboPol-21 (Struers). The Lambda detector was placed a few centimetres behind the exit window of the flight tube. The distance between the focal plane and Lambda was measured to be 5.07 m.

### 2.2. Samples   

**Figure 2:**
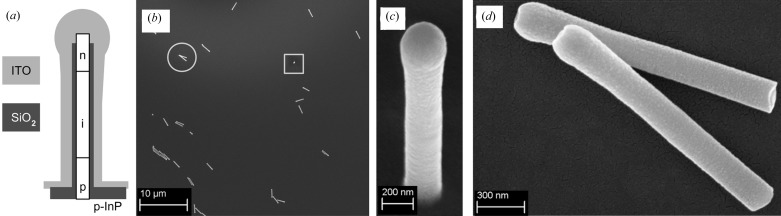
(*a*) Schematic of a nanowire solar cell consisting of InP substrate (p-type at the bottom and n-type on top), an insulating SiO_2_ layer and an ITO cover. (*b*) shows an SEM image of the nanowires deposited on the Si_3_N_4_ membrane after the X-ray experiment. (*c*) is an SEM image of the standing nanowire in (*b*) which is highlighted by a grey rectangle. The view is at an angle of 45°. (*d*) shows two lying nanowires of the sample [*cf.* circle in (*b*)]. Here, the diameter was measured to be about 300 nm at the body and 360 nm at the head. The length is about 2.6 µm.

The nanowires in this work (Fig. 2 ▶) were grown and processed as described in Wallentin *et al.* (2013 ▶) and Wallentin (2013 ▶). Briefly, (i) gold seed particles were formed on an InP substrate by nano-imprint lithography (Mårtensson *et al.*, 2004 ▶), (ii) InP nanowires were grown with vapour-phase epitaxy, (iii) the gold was removed by wet etching, (iv) SiO_2_ deposition (insulator) by atomic layer deposition and (v) sputter deposition of an optically transparent and conducting layer of indium tin oxide (ITO). After fabrication, the nanowires were cut off the substrate by gently wiping the surface with a soft tissue. Nanowires on the tissue were then allowed to adhere on an Si_3_N_4_ membrane (1 µm thickness) (Silson, UK). This process yields randomly distributed nanowires (Fig. 2 ▶ *b*), which occasionally are oriented perpendicular to the membrane (Fig. 2 ▶ *c*). After the X-ray experiment the sample was sputtered with a 5 nm gold layer for scanning electron microscopy (SEM) imaging.

### 2.3. Data recording and data treatment   

At first, the attenuation profile of the STCS was measured with the Lambda by using an open slit setting of S1 = S2 = 300 × 300 µm and sufficient beam attenuation (Fig. 1 ▶ *b*). The rescaling of STCS-attenuated diffraction patterns is exemplified in Fig. 1 ▶(*c*).

The first ptychographic scan (data set 1) covers an area of 3 × 3 µm around the nanowire that was oriented perpendicular to the membrane or ‘standing’ (Fig. 2 ▶ *c*). Here, 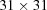 scan points were taken on a rectangular grid with step sizes of 100 nm. At each point the exposure time was 1 s. Including movements of motors and readout, the total scanning time was about 29 min. The scan of data set 2 was recorded on a nanowire being oriented parallel to the membrane (‘lying’) with the same parameters as data set 1, except only 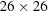 scan points were distributed equidistantly over a square of 2.5 × 2.5 µm. For both data sets a small beam-defining slit gap S1 of 50 µm was chosen and the photon flux impinging on the sample was reduced by molybdenum foils to about 10^7^ photons s^−1^ (*cf.* Table 1 ▶). The ptychographic data set 3 was taken on the same nanowire with identical scan parameters as data set 2. The total scanning time was about 20 min in both cases. In contrast, the beam-defining slit gap S1 was opened to 100 µm, which corresponds to an increase in flux by a factor of about 4. Importantly, no beam attenuation in front of the sample was used and the higher photon flux (increase by a factor of 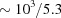) of the KB beam on the detector was compensated by inserting the STCS in front of the Lambda detector.

The design of the Lambda detector is such that the gaps between the chips are filled with pixels larger than the chip pixels (55 × 55 µm). There are 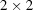 pixels in the intersections of horizontal and vertical gaps with a size of 165 × 165 µm. The remaining pixels in the gaps are 55 × 165 µm (h × v) and 165 × 55 µm (h × v) in the horizontal and the vertical gaps, respectively. The data in the gap pixels were rebinned to the size of the chip pixels.

The ptychographic reconstruction for both object and probe function was carried out using the ‘ePIE’ algorithm (using 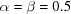; *cf.* Maiden & Rodenburg, 2009 ▶). The algorithmic settings are the same for all three data sets. The reconstruction procedure was initialized using a Gaussian (FWHM = 200 nm) amplitude distribution as probe guess and a unity, pure amplitude plane as object. The number of ptychographic iterations was 600. The final results of the probe and object were obtained by averaging the current estimate over the last 200 iterations.

## 3. Results   

### 3.1. Ptychographic reconstruction of KB wavefields   

**Figure 3:**
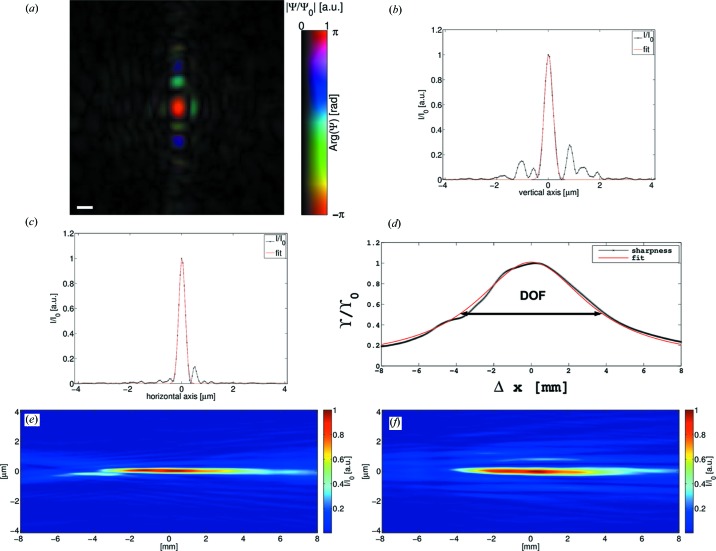
(*a*) Complex field of the probe of the low-photon-flux data set 1 (*cf.* Table 1 ▶ and Fig. 5 ▶) propagated to the focal plane. Amplitude and phase are drawn according to the colour bar next to the image. In (*b*) and (*c*) line cuts through the vertical and horizontal direction of the probe intensity in the focus (*a*) are drawn, respectively. Gaussian fits to the curves (red lines) yield FWHM = 393 nm and FWHM = 292 nm in (*b*) and (*c*), respectively. (*d*) Sharpness and fit for estimation of the DOF. (*e*) shows the horizontal slice of the probe intensity through the focus. (*f*) presents the vertical slice. The scale bar in (*a*) denotes 500 nm.

The results of the ptychographically reconstructed complex wavefield incident on the sample of data set 1 (S1 gap 50 µm) are presented in Fig. 3 ▶. In a first step, the reconstructed probe was numerically propagated along the optical axis by 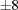
 mm. The focal plane was determined to be 240 µm upstream of the sample by using the ‘sharpness’ criterion (*e.g.* Guizar-Sicairos, 2010 ▶): 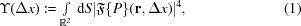
where 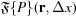 denotes the free space propagation of the reconstructed probe field *P* by 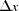. The maximum of 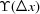 determines the focal plane. The complex wavefield of the focal plane is shown in Fig. 3 ▶(*a*). It can be seen that side lobes are more pronounced in the vertical direction. The phase representation in the image reveals an effectively flat phase of the central peak. Line cuts through the maximum of the peak can be seen in Figs. 3 ▶(*b*), 3 ▶(*c*). Here, the difference in side-lobe formation between the horizontal and vertical direction is visible, too. Using a Gaussian fit to determine the size of the focus yields FWHM = 292 nm in the horizontal direction. For the vertical direction FWHM = 393 nm is obtained. A horizontal and a vertical slice through the focus and along the optical axis is shown in Figs. 3 ▶(*e*) and 3 ▶(*f*), respectively. The depth of focus (DOF) extends visibly over a few millimetres. The ‘sharpness’ can also be used to quantify the DOF. In the case of a Gaussian intensity distribution 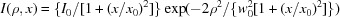
along the radial direction 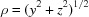 with Rayleigh length 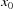 and beam waist 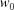 the sharpness can be analytically expressed: 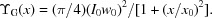
Therefore, the normalized sharpness 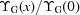 is a measure of the Rayleigh length 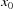 of the beam, which is equal to half of the DOF. A fit of 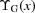 to the sharpness of the re­con­structed probe field yields DOF = 2*x*_0_ = 7.32 mm (*cf*. Fig. 3 ▶ *d*).

**Figure 4:**
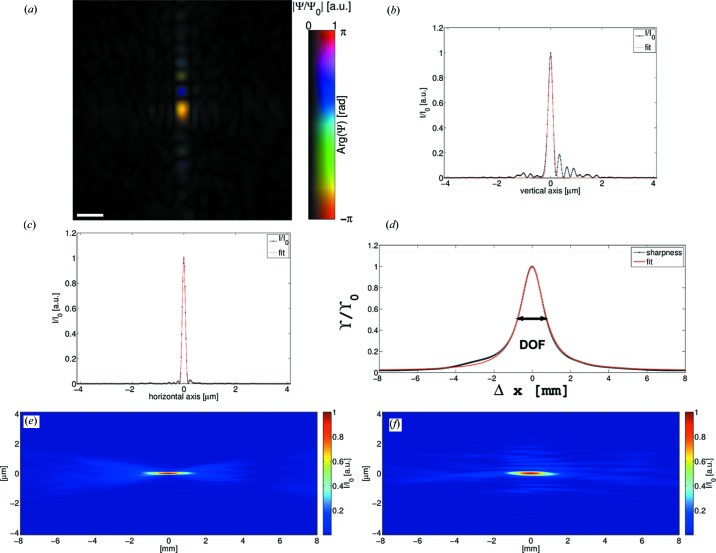
(*a*) Complex field of the probe of the high-photon-flux data set 3 (*cf.* Table 1 ▶ and Fig. 6 ▶) propagated to the focal plane. Amplitude and phase are drawn according to the colour bar next to the image. In (*b*) and (*c*) line cuts through the vertical and horizontal direction of the probe intensity in the focus (*a*) are drawn, respectively. Gaussian fits to the curves (red lines) yield FWHM = 217 nm and FWHM = 136 nm in (*b*) and (*c*), respectively. (*d*) Sharpness and fit for estimation of the DOF. (*e*) shows the horizontal slice of the probe intensity through the focus. (*f*) presents the vertical slice. The scale bar in (*a*) denotes 500 nm.

The reconstructed wavefield of data set 3 (S1 gap 100 µm) is analysed in the same way. In this case, the focal plane almost coincides with the plane of the sample. The distance between sample plane and focal plane is determined to be 16 µm. The intensity distribution of the focal plane reveals a smaller peak with reduced side lobes (*cf.* Fig. 4 ▶ *a*). In the line cuts through the central peak it can be seen that the intensity of the side lobes is reduced by a factor of about 2 in comparison to the other probe field. Gaussian fits to the curves yield a beam width of FWHM = 217 nm and FWHM = 136 nm in the vertical (Fig. 4 ▶ *b*) and horizontal (Fig. 4 ▶ *c*) direction, respectively. Hence, the decrease in size is by a factor of 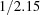 in the horizontal direction and by a factor of 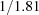 in the vertical direction. Moreover, the decrease in size of the beam can also be observed in the slices through the focus and along the optical axis as a decrease in the depth of focus (*cf.* Figs. 4 ▶ *e*, 4 ▶ *f*). A fit of 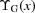 to the sharpness of the reconstructed probe field yields DOF = 2*x*_0_ = 1.52 mm (*cf.* Fig. 4 ▶ *d*).

### 3.2. Ptychographic imaging of nanowires   

**Figure 5:**
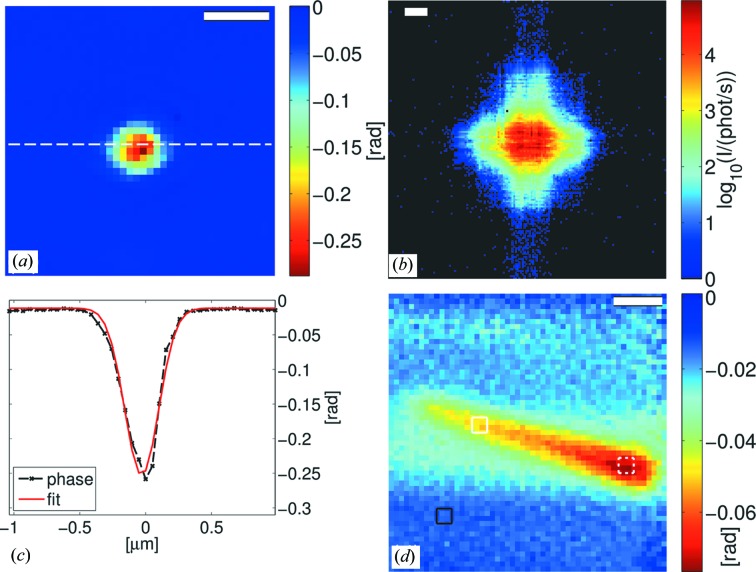
(*a*) Phase reconstruction of the standing nanowire (data set 1, Fig. 2 ▶ *c*). (*b*) shows a diffraction pattern of data set 1. (*c*) presents a line cut through the phase reconstruction shown in (*a*) (black dashed line). A fit of a Gaussian function (red line) to the phase profile of the nanowire yields an FWHM value of 305 nm. (*d*) presents the phase reconstruction of a lying nanowire (data set 2). The thicker top of the nanowire (*cf.* Figs. 2 ▶ *a*, 2 ▶ *c*) can be clearly identified by a larger phase shift which appears red in the image. The nanowire yields an average phase shift of 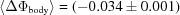
 rad on the body (dashed white frame) and 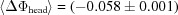
 rad on the head (dash–dotted white frame) after background subtraction (dashed black frame). Scale bars in (*a*) and (*d*) denote 500 nm, whereas the scale bar in (*b*) depicts 10 µm^−1^.

At first, we list the results of the ptychographic reconstructions using a (coherent) photon flux of about 10^7^ photons s^−1^ and a small beam-defining slit gap S1 of 50 µm (Fig. 5 ▶). The image of the reconstructed phase of data set 1 (Fig. 5 ▶ *a*) shows the phase shift of the standing nanowire (oriented perpendicular with the membrane, Fig. 2 ▶ *c*). A line cut through the phase reconstruction of the nanostructure is drawn in Fig. 5 ▶(*c*). It is indicated in the original figure as a dashed white line. The maximum phase shift of the nano­structure in this graph is about −0.24 rad (after background subtraction). A Gaussian fit to the curve yields a width estimate of 305 nm (FWHM). A typical diffraction pattern of the KB beam imprint on the detector of this data set is shown in Fig. 5 ▶(*b*). The maximum count rate in the diffraction pattern is 91 442 photons s^−1^ per pixel. It should be noted that the count rate is still below the maximum count rate of 3 × 10^5^ photons s^−1^ per pixel of the Lambda detector. In Fig. 5 ▶(*d*) the phase of the ptychographic reconstruction of a lying nanowire (oriented parallel to the membrane) can be clearly seen. The length is about 2.6 µm. The extent of the nanowire along the minor axis is in the range of 400–500 nm. The phase shift was estimated from the reconstruction by averaging the phase map over small regions. An offset phase (black frame) was subtracted. On the body of the nanowire the average phase shift yields 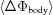 = 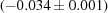
 rad (white frame) and on the central part of the head the phase shift is increased in magnitude to 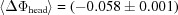
 rad. The pixel size of both reconstructions is 52 nm.

**Figure 6:**
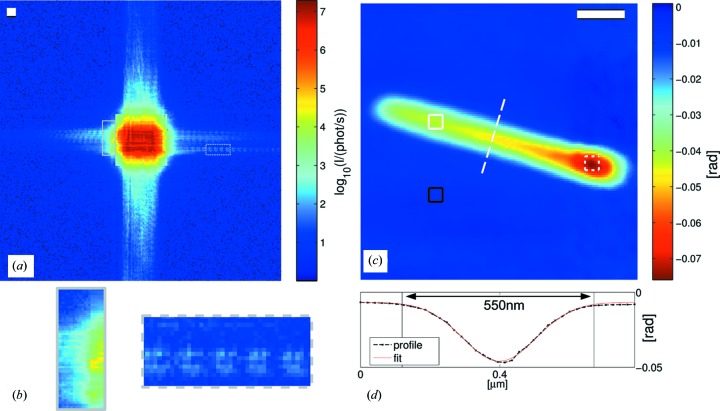
(*a*) shows a diffraction pattern of data set 3. The central part of the diffraction pattern has already been rescaled by the attenuation mask of the STCS. (*b*) shows enlarged parts of the diffraction pattern in (*a*). The intermediate region between adjacent chips where the remapping of the recorded intensity from larger to smaller pixels yields good results due to high photon count rates can be seen on the region with a solid grey frame. The other region highlights the high-frequency oscillations of the KB farfield on the detector (dashed grey frame). (*c*) presents the recontructed phase of the lying nanowire corresponding to the data recording using the STCS (data set 3). The nanowire is the same as in data set 2 (Fig. 5 ▶ *d*). It yields an average phase shift of 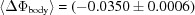
 rad on the body (white frame) and 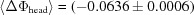
 rad on the head (dashed white frame) after background subtraction (black frame). (*d*) shows the line profile in (*c*) as indicated by a dashed white line. A Gaussian (FWHM = 247 nm) is well fitted to the profile. Scale bars in (*a*) and (*c*) denote 10 µm^−1^ and 500 nm, respectively.

Next, we consider the result of the ptychographic reconstructions using a (coherent) photon flux of 9.8 × 10^9^ photons s^−1^ and a beam-defining slit gap S1 of 100 µm (Fig. 6 ▶). A representative diffraction pattern (after correction for the STCS attenuation) of data set 3 is shown in Fig. 6 ▶(*a*). In this setting the Lambda detector can record the diffracted intensity over more than seven orders of magnitude. The KB imprint is obviously larger in comparison to the small S1 gap setting (*cf.* Fig. 5 ▶ *b*). It can be seen that borders of the central part of the beam have been placed close to the intermediate regions between neighbouring chips of the Lambda. Although rescaled, the regions between the chips can be identified in regions of low count rates. However, a smooth rescaling can be obtained if the count rate is sufficiently high. For instance, this can be seen where the tails of the KB beam extend over two chips (*cf.* Fig. 6 ▶ *b*). In addition, the high dynamic range of the signal reveals the fine features of the tails of the KB beam whose signal lies three to seven orders of magnitude below the central beam signal. Small oscillations in the range of a few pixels are characteristic and are highlighted by an enlarged representation (*cf.* Fig. 6 ▶ *b*).

The phase reconstruction of data set 3 shows the lying nanowire of data set 2 (Fig. 6 ▶ *c*). Here, the pixel size is 21 nm. In comparison to the reconstruction of data set 2, the nanowire appears smoother with less background. The resolution is visibly improved. In particular, the shape of the head of the nanowire resembles more the shape that is observed in the SEM images (Fig. 2 ▶). A quantification of the resolution follows in the next section. The measured phase shift on parts of the head (dashed, white frame) and the body (white frame) are 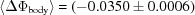
 rad and 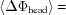 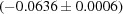
 rad, respectively. A line profile along the minor axis indicates the thickness of the nanowire (Fig. 6 ▶ *d*). The shape is well fitted by a Gaussian function with an FWHM = 247 nm.

## 4. Discussion, conclusion and outlook   

Firstly, we discuss the ptychographic reconstructions of the wavefields. The probe reconstruction allows us to compare the KB wavefield between two different optical settings. A change of the slit gap setting causes a change of the size of the KB focus (Matsuyama *et al.*, 2006 ▶). Here, the increase of the gap width of the beam-defining slit by a factor of 2 could be shown to result in a decrease in size of the focus approximately by a factor of 2. The result is in agreement with Fourier optics where the focus size decreases linearly with an increase of the numerical aperture indicating full or a high degree of spatial coherence of the beam. In comparison to preceding experiments (Giewekemeyer *et al.*, 2013 ▶, 2014 ▶; Wilke *et al.*, 2013 ▶) where typically a pinhole was used in front of the focal plane, we can show here the effectively pure KB beam, which is only slightly cleaned by the soft-edge aperture. Moreover, the application of the STCS makes it possible to reconstruct the probe from high-dynamic-range intensity distributions, which cover about seven orders of magnitude. The photon flux of 9.8 × 10^9^ photons s^−1^ is still below the maximum coherent flux of about 10^11^ photons s^−1^ for larger slit gaps at the GINIX (*cf.* Salditt *et al.*, 2011 ▶) and thus limited by the geometric dimensions of the STCS which define the maximum slit gaps (*cf.* Fig. 1 ▶ *b*).

Next, the ptychographic reconstructions of the nanowires need to be considered. The phase shift of the lying nanowire should be comparable in both data sets 2 and 3 (Figs. 5 ▶ *d*, 6 ▶ *c*). Indeed, we find a good agreement of the overall phase shift between both data sets. Moreover, the average phase shift over small parts of the body deviates less than 4% between both data sets. The slight deviation in the head region can be explained by the fact that the phase shift of a rod or cylindrically shaped object is not homogeneous, which makes it difficult to define an average phase shift. In brief, the reconstructions are very consistent. The agreement in phase shift between the data sets holds also for the projection of the standing nanowire. From the SEM images we infer a length of about 2.1 µm and a thickness of about 0.3 µm. Using the phase shift as determined from the ptychographic reconstruction of data set 3, an expected phase shift of 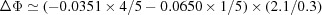
 rad = −0.29 rad is obtained for a model nanowire that consists of 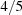 of body and 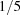 of head (both of thickness 0.3 µm), which is in good agreement with the reconstruction (Fig. 5 ▶ *a*). However, we note a discrepancy in comparison to tabulated values. For instance, the typical width of the InP core of the nanowires shown here is not below 150 nm, which already yields a phase shift of −0.048 rad (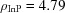
 g cm^−3^) at 13.8 keV (Henke *et al.*, 1993 ▶). It is known that a wrong rescaling of the STCS can induce errors in the phase shift (Wilke *et al.*, 2013 ▶). On the other hand, the consistency of the two phase maps (with and without STCS) is a good quality control of the STCS rescaling. Therefore we are confident about the quantitativeness of the ptychographic reconstructions with respect to the STCS rescaling.

**Figure 7:**
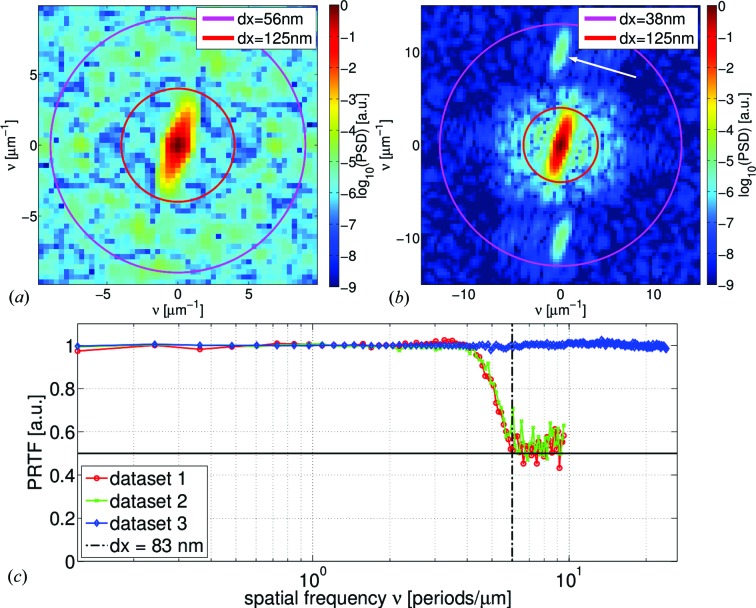
(*a*) and (*b*) show two-dimensional power spectral densities of the phase reconstructions of data sets 2 and 3 (*cf.* Figs. 5 ▶ *d* and 6 ▶ *c*), respectively. The frequency contributions from the rod shape of the nanowire lie within a frequency ring that corresponds to 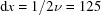
 nm half-period resolution. In the power spectrum of data set 3 (*b*) frequency contributions appear also in the frequency shell corresponding to 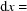 38–125 nm (*cf.* white arrow). (*c*) presents PRTF calculations of the three data sets: 1 (red), 2 (green), 3 (blue). The drop of the PRTF below 0.5 (horizontal solid black line) is indicated by a vertical dash–dotted black line at 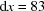
 nm half-period resolution.

Finally, the improvement of resolution due to the gain in photon flux from data set 2 to 3 needs to be addressed. Here we could achieve a visible improvement of the reconstruction in terms of resolution and background. However, we also note that the diameter of the nanowire in the reconstruction is larger than expected from SEM images (Fig. 2 ▶) that give about 300–350 nm. A comparison of the two-dimensional power spectral densities (PSDs) of the reconstructed phase maps (*cf.* Fig. 7 ▶) reveals that most information on the nanowire (the shape) is contained within a frequency ring that corresponds to a resolution of about 125 nm. In the case of data set 3, the inner frequency part appears to be slightly larger but there are also contributions for structure sizes of 38 nm (*cf.* white arrow in Fig. 7 ▶ *b*). Another estimate of the resolution that also estimates the correctness of the phasing of the algorithm can be obtained by calculating the so-called ‘phase-retrieval transfer function’ (PRTF) (*cf.* Shapiro *et al.*, 2005 ▶; Chapman *et al.*, 2006 ▶; Giewekemeyer *et al.*, 2010 ▶): 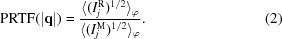
Here, 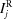 and 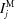 denote the reconstructed and measured intensity at position *j* (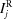 corresponds to the object and probe being averaged over the last iterations), respectively. 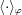 indicates azimuthal averaging, * i.e.* averaging over 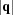 = 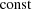. The PRTFs of data set 1 (red) and 2 (green) are very similar. Both curves drop below 0.5 at about 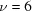
 µm^−1^ (half-period) indicating that the resolution of these data sets is not better than 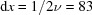
 nm. In contrast, the PRTF of data set 3 (blue) remains close to unity without any significant descent. It should be noted that this only indicates a resolution of the probe field in the range of the pixel size (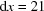
 nm), *i.e.* the contributions of the object are ‘hidden’ (Dierolf *et al.*, 2010 ▶; Giewekemeyer *et al.*, 2010 ▶). The resolution of the object cannot be better than 38 nm as the PSD analysis revealed. As can be seen by comparing the images in Figs. 5 ▶(*d*) and 6 ▶(*c*), little more information about the structure of the nanowires could be obtained. For instance, in the optimal case one would be able to observe a deviation from the smooth Gaussian curve across the nanowire rod (*cf.* Fig. 6 ▶ *d*) due to the different layers of material.

Next, let us compare the increase in fluence, which can be used to estimate the potential gain in resolution due to the increase in dose. The dose *D* depends on the photon energy *E*, the linear absorption coefficient μ, the mass density 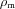 and the fluence *F* (Howells *et al.*, 2009 ▶):
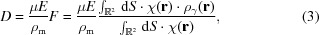
where 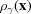 denotes the two-dimensional photon density and 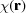 is the characteristic function of the probed area. In order to account for the overlap of the ptychographic data 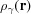 is obtained by scaling the normalized intensity of the probe 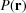 at each scan position *j* to the actual photon number 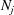 (Wilke *et al.*, 2012 ▶):
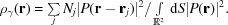
Here we define the fluence on the sample per single recorded diffraction pattern by taking only the photons of the reconstructed single probe intensity 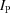 around the central peak into account, *i.e.* 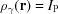 and χ corresponds to an ellipse whose minor and major axes are defined by the half width at half-maximum (FWHM/2) of the intensity distribution. The fluence of the low-flux data set 2 is estimated to be 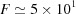
 photons nm^−2^ and the high-flux data set 3 yields 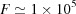
 photons nm^−2^ (equivalent to a flux density of 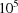
 photons nm^−2^ s^−1^). On the other hand, the overall average fluence over the area of the sample which accounts for the overlap [equation (3)] yields an even higher fluence of 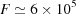
 photons nm^−2^ for data set 3. Hence, the STCS in combination with the beam resizing allows the fluence on the sample to be increased by three to four orders of magnitude. At first, we note that the fluence obtained here is quite high. For instance, 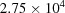
 photons nm^−2^ has been reported by Schropp *et al.* (2012 ▶) using 15.25 keV X-rays focused down to ∼80 × 80 nm (smaller by a factor of 5). Schropp and co-workers estimated their resolution on a 500 m-thick binary tantalum test structure to be about 10 nm. Hence, at least on test structures with sharp edges, the potential resolution should be in the range of 10 nm, which is in good agreement with preceding experiments (Wilke *et al.*, 2013 ▶). One way of explaining the discrepancy between expected and observed resolution would be to attribute the result to non-ideal experimental parameters such as the effects of partial coherence. However, the PRTF of data set 3 indicates that at least the probe reconstruction appears to be very good up to the highest frequency. Hence, vibrations of the sample as discussed by *e.g.* Clark *et al.* (2011 ▶) may explain our results, also with respect to the observed deviation of the phase shift of the nanowire from tabulated values. Another degrading factor could be fluorescence radiation from the Ge STCS. In addition, a supposedly minor effect may be small sensitivity variations of the pixels of the Lambda detector (Schavkan *et al.*, 2013 ▶), which could be compensated by a flatfield correction.

In conclusion, we have shown a powerful ptychographical experimental setting that paves the way for experiments with unprecedented flux density. We have presented first ptychographic reconstructions using the new Lambda detector. The use of a higher energy of 13.8 keV is an improvement, as future experiments investigating elemental constituents of the sample will take advantage of the wider range of available *K* edges such as Ni, Cu, Zn and Br in comparison to earlier experiments using 7.9 keV. In addition, the reconstructions clearly benefit from the effectively non-missing data problem between adjacent chips of the detector, which is a common problem for many other pixel detectors. The high count rate of the Lambda detector in combination with the STCS and the reconstruction of the probe field has enabled us to set up a fluence-optimized ptychographic setting. As far as we could deduce from other works in this field, the flux density of about 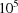
 photons nm^−2^ s^−1^ is the highest one that has been reported at synchrotron storage rings for ptychographic imaging experiments in combination with photon-counting pixel detectors (*cf.* Table 2 ▶). In addition, there are two more features of the Lambda detector that have not been addressed in these experiments but which may become important for following experiments. Firstly, the small pixel size of 55 µm in comparison to *e.g.* 172 µm (Pilatus, Dectris) relaxes oversampling requirements. In simple terms, oversampling limits the ratio between beam size and field of view (FOV) in the sample plane per single diffraction pattern (Miao *et al.*, 1998 ▶). According to the small-angle approximation the FOV per single diffraction pattern depends on the wavelength λ, the distance between sample and detector *X* and the pixel size of the detector *D*, *i.e.* 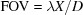. The smaller pixel size thus allows a large field of view (here ∼8 × 8 µm), which in principle opens the possibility for large beam experiments. Consequently, the total scanned area of the sample can be increased while the number of scan points is kept constant. The small pixel size also alleviates the use of higher energies (or smaller distances between sample and detector). Secondly, the 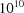
 photons s^−1^ of the beam can also be distributed in favour of shorter exposure times. Thus reduced scanning times would presumably increase the quality of reconstructions due to a decrease of thermal drifts and vibrations. The Lambda can be operated in a continuous read–write mode and it provides a read-out of about 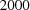
 frames s^−1^. Hence, the detector is very well suited for short exposure times, and it may become important in experiments where deadtime is crucial.

## Figures and Tables

**Table 1:** Information on used slit settings

Data set	Slits	Horizontal gap (m)	Vertical gap (m)	Photon flux (photonss^1^)
1	S1	50	50	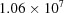
	S2	200	200	
2	S1	50	50	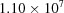
	S2	200	200	
3	S1	100	100	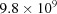
	S2	200	200	

**Table 2:** Flux-density achievements in ptychographic imaging experiments Estimations have been obtained according to the reported beam size (see text).

Flux density (photonsnm^2^s^1^)	Beamline	Reference
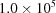	P10 PETRAIII	This work
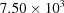	P10 PETRAIII	Giewekemeyer *et al.* (2014 ▶)
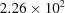	P10 PETRAIII	Wilke *et al.* (2013 ▶)
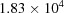	P06 PETRAIII	Schropp *et al.* (2012 ▶)
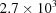	cSAXS SLS	Guizar-Sicairos *et al.* (2012 ▶)
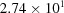	BL29XUL SPring-8	Takahashi *et al.* (2011 ▶)
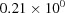	cSAXS SLS	Giewekemeyer *et al.* (2010 ▶)
